# Development of Efficient Supramolecular Photostabilizer for Carotenoids and Retinoids: Analyses and Application Research

**DOI:** 10.1111/jocd.70650

**Published:** 2026-01-02

**Authors:** Mengqing Zhao, Shuang Chen, Ke Hu, V. I. Evseenko, E. S. Meteleva, M. V. Zelikman, A. V. Dushkin, A. V. Mastova, M. A. Ulyanova, P. A. Kononova, O. Yu. Selyutina, Wenhao Xu, N. E. Polyakov

**Affiliations:** ^1^ Collaborative Innovation Center of Yangtze River Delta Region Green Pharmaceuticals Zhejiang University of Technology Hangzhou China; ^2^ Zhejiang Jingxin Pharmaceutical co., Ltd. Shaoxing China; ^3^ Institute of Solid‐State Chemistry and Mechanochemistry Novosibirsk Russia; ^4^ Institute of Chemical Kinetics and Combustion Novosibirsk Russia

**Keywords:** carotenoids, crocin, glycyrrhizin, mixed micelles, photostabilizer, photo‐protection, retinol

## Abstract

**Background:**

Carotenoids and retinol are well known for their potent antioxidant and biological properties in pharmaceuticals, foods, and cosmetics. However, their poor photostability presents a significant challenge that cannot be effectively addressed through simple formulation strategies such as encapsulation. Although photostabilizers are commonly employed to enhance product photostability, there remains a lack of highly water‐soluble and legitimate stabilizers suitable for commercial cosmetic applications.

**Aims:**

To develop a novel aqueous‐phase photostabilizer simultaneously boosting carotenoid and retinol shelf‐life in cosmetics and meeting global safety standards. Underlying mechanism and application are investigated.

**Methods:**

Micellar supramolecular complex crocin–Na_2_GA was prepared by mechanochemistry; thermal stability, storage stability, and photo‐oxidative degradation were tested. Then, photoprotection of carotenoids and retinoids by crocin–Na_2_GA in aqua solution, cream‐based formulations, and on an artificial skin model was tested. Finally, cyclic voltammetry was applied for mechanism research.

**Results:**

Experiments demonstrated that the light‐induced degradation of astaxanthin, β‐carotene, and retinol in aqueous solutions, cream formulations, and on skin was significantly reduced upon the addition of crocin–Na_2_GA. These findings indicated that crocin–Na_2_GA possessed remarkable photoprotective properties. Mechanistic studies further revealed that within the stable nano micellar system of crocin–Na_2_GA, crocin was readily photoactivated and scavenged photoinduced radicals through a self‐sacrificial mechanism, thereby preventing the photodegradation of active constituents during the storage and application phases of creams.

**Conclusions:**

Collectively, these results highlight the potential of crocin–Na_2_GA as a highly efficient, safe, and cost‐effective photostabilizer, making it a promising candidate for incorporation into cosmetic formulations.

## 1 Introduction

Carotenoids and retinoids are essential functional compounds widely used in pharmaceuticals, foods, and cosmetics. They exhibit potent biological activities, acting as free radical scavengers, whitening agents, as well as anti‐acne and anti‐wrinkle agents [1]. However, these substances exhibit significant photosensitivity issues during application and are vulnerable to oxidative degradation caused by photoinduced oxygen radicals. This process leads to the decomposition of active ingredients and the formation of allergenic byproducts [2]. To prevent the photodegradation and potential phototoxicity of carotenoids and retinoids, formulations are often supplemented with chemical UV absorbers containing diphenyl ketones or hindered amines as their core structural units [3]. However, these substances exhibit notable toxicity [4, 5] and are therefore prohibited for use in pharmaceutical, cosmetic, and food products. Furthermore, currently available light stabilizers possess weaker reducing properties compared to conjugated polyenes [6, 7, 8]. Consequently, polyene‐like carotenoids tend to react readily with photoinduced radicals, making it difficult to achieve effective photostabilization at safe concentrations. Therefore, it is essential to develop effective, safe, and edible photostabilizers for carotenoid‐ and retinoid‐based pharmaceuticals, foods, and cosmetics. These stabilizers must exhibit stronger free radical quenching capabilities compared to carotenoids themselves. However, there remains a scarcity of photostabilizers that combine safety, cost‐effectiveness, and efficiency, resulting in increased production costs and reduced efficacy.

In recent years, considerable efforts have been directed toward developing novel carotenoid and retinoid complexes to improve their stability. In these studies, carotenoids and retinoids are typically encapsulated by or absorbed onto inorganic, synthetic, or natural polymers to form complexes such as mixed nanoparticles [9, 10, 11, 12], micelles, microcapsules [13, 14, 15], or molecular complexes in solution [16]. These complexes can effectively protect the conjugated double bonds in carotenoids and retinoids from environmental factors, improving their thermal and storage stability by 20%–30%. However, because photons are extremely small and exhibit wave–particle duality, encapsulation, mixed micelle formation, and inorganic adsorption cannot completely prevent photon penetration, inhibit photorefractory generation, or suppress the formation of photoinduced radicals. In summary, poor light stability remains the most significant challenge limiting the application of carotenoid‐ and retinol‐based formulations, and existing technologies have not adequately addressed this issue.

Our previous study [17] revealed a notable finding that the micellar supramolecular complex formed by crocin and the natural saponin disodium glycyrrhizinate (Na_2_GA) exhibits rapid crocin degradation upon light exposure, at a rate more than 10 times faster than that observed under dark conditions. This process is unaffected by heat or oxidants. Although the presence of multiple conjugated double bonds in crocin contributes to its free radical scavenging activity [18, 19, 20, 21], its amphiphilic nature promotes its aggregation in solution, where sugar moieties cluster at both ends. This aggregation limits the interaction between crocin and its surrounding environment [19], resulting in antioxidant activity weaker than that of other carotenoids. However, this serendipitous finding suggests that the crocin–Na_2_GA mixed micellar system can significantly enhance the ability of crocin to scavenge photoinduced radicals, indicating its potential as a self‐sacrificial photostabilizer.

Moreover, this system offers several advantages for commercial light‐stabilizing applications. Both crocin and Na_2_GA are approved additives that are inexpensive, highly water‐soluble, and safe for use in pharmaceuticals, cosmetics, and foods. They also exhibit multiple biological functions, including anti‐inflammatory, antibacterial, antiviral, antidepressant, and soothing effects. Additionally, the surface activity of Na_2_GA [22, 23, 24, 25] enables it to stabilize lipophilic compounds and enhance their permeability through the lipid bilayer of enterocytes. It should be emphasized that crocin and Na_2_GA have been established as safe and functional adjuvants for pharmaceutical, cosmetic, and food use [26, 27, 28]. Therefore, these findings raise the question of whether crocin–Na_2_GA micelles can serve as safe and efficient self‐scavenging light stabilizers in such products.

Inspired by the unexpected photoinstability observed in the crocin–Na_2_GA supramolecular complex, we conducted a comprehensive investigation into crocin‐based micellar systems. The study had two primary objectives: first, to explore the role of the system as a self‐sacrificial photoprotective agent, and second, to evaluate its function as a stabilizing agent for formulations containing carotenoids and retinols as active ingredients. Detailed assessments of the system's stabilizing effects and underlying mechanisms were also conducted, followed by its application in cosmetic formulations. These findings represent a significant effort to leverage the antioxidant properties of crocin to develop safer and more effective light stabilizers for the pharmaceutical, cosmetic, and food industries, underscoring the importance of this study.

## 2 Materials and Methods

### 2.1 Materials

Crocin was purchased from Suichang Limin Pharmaceutical Co. Ltd. (China). Na_2_GA (99.5% purity) and Arabinogalactan (AG) were obtained from Shaanxi Panier Biotechnology Co. Ltd. (China). Ferric chloride (FeCl_3_ · H_2_O, AR, > 99% purity), lithium nitrate (LiNO_3_, AR, > 99% purity), sodium azide (NaN_3_, AR, > 99% purity), and glycerin (AR, > 99% purity) were purchased from Yuanye Biotechnology Co. Ltd. (China). Astaxanthin (> 10% purity, water soluble, the emulsifier is lecithin) was obtained from Shaanxi Guanchen Biotechnology Co. Ltd. (China), and β‐carotene (> 10% purity, water soluble, the emulsifier is lecithin) was supplied by Hubei Xinhe Biotechnology Co. Ltd. (China). Retinol was obtained from Beijing Beilis Biotechnology Co. Ltd. (China, > 10% purity, water soluble, the solubilizing excipients are hydroxypropyl cyclodextrin and 1,3‐butanediol). Olive oil was purchased from Shanghai Kerry Food Industries Co. Ltd. (China). The compound emulsifier SETHIGEL G57 was sourced from Sisley (France). Crocin supramolecular complexes, including crocin–Na_2_GA and the control complex crocin–AG, were prepared and optimized according to the method described in the Supporting Information.

### 2.2 Analysis of Compound Concentrations

To determine the concentrations of crocin, astaxanthin, retinol, and β‐carotene in the complexes, 5 mg of each sample was dissolved in 5 mL of water. After complete dissolution and filtration, the solution was diluted and analyzed by high‐performance liquid chromatography (HPLC). The analysis was performed using an Agilent 1200 HPLC system (Agilent, USA) equipped with a Zorbax Eclipse XDB C_18_ reversed‐phase column (5 μm, 4.6 × 50 mm, Agilent, USA). The mobile phase consisted of methanol and a 0.05% aqueous acetic acid solution at a volume ratio of 6:4. The crocin concentration was measured at 30°C using a detection wavelength of 440 nm, a flow rate of 0.8 mL/min, and an injection volume of 5 μL.

### 2.3 Particle Characterization and Molecular Weight Distribution Analysis

Physicochemical characterization was conducted at 25°C using a Zetasizer NanoZS instrument (Malvern Instruments, UK). Sample solutions were prepared by dissolving the materials in deionized water to a final concentration of 1 mg/mL. The particle size, polydispersity index (PDI), and surface charge (ζ‐potential) of the nanomicelles were measured using dynamic light scattering (DLS) and laser Doppler anemometry. The morphology of crocin–Na_2_GA in aqueous solution was observed using transmission electron microscopy (TEM, JEM‐2100EX, JEOL, Japan) at an accelerating voltage of 100 kV. TEM images were obtained and analyzed on the first day and after 10 days of storage.

The molecular weight distribution of each sample was determined by dissolving crocin in an aqueous solution containing 0.2% NaN_3_ and 0.5 M LiNO_3_, followed by size‐exclusion HPLC analysis at a flow rate of 1 mL/min and a column temperature of 30°C. Calibration was performed using dextran standards with molecular weights of 1, 5, 12, 25, 80, 150, 270, and 410 kDa. Data were processed using GPC/SEC software (Agilent, USA).

### 2.4 Analysis of Crocin Photo‐Oxidative Degradation

UV–visible spectroscopic analysis was performed using an SF‐2000 UV–visible spectrophotometer (Spectrum, Russia) equipped with 1 cm and 0.2 cm quartz cuvettes. For the preparation of stock solutions, crocin or its complexes (1 mM crocin) were dissolved in H_2_O. To study the oxidation kinetics, the concentrated solutions were diluted to 0.01 or 0.1 mM in a quartz cuvette, followed by the addition of FeCl_3_ (1 mM). The reaction was monitored by recording the changes in the absorption spectrum of crocin at λ = 440 nm (λ_max_ for crocin).

The photo‐oxidative degradation of crocin was then studied. It is well known that photogenerated oxygen radicals, particularly hydroxyl radicals, can disrupt polyconjugated molecular structures and induce their photo‐oxidative degradation. This process can be further accelerated in the presence of iron ions. In this study, both photo‐oxidative degradation and Fe‐catalyzed photo‐oxidative degradation of crocin and its complexes were investigated.

For the photo‐oxidation resistance test, aqueous solutions of crocin, crocin–AG (Arabinogalactan is short for AG), and crocin–Na_2_GA with fixed crocin concentrations (0.1 and 0.01 mM) were prepared and irradiated using a Lambda Physik EMG 101 MSC excimer laser (308 nm, 100 mJ/pulse) and a mercury lamp (DRSh‐500, 500 W, full‐spectrum light passed through a water filter). The samples were irradiated in standard 10 and 2 mm quartz cuvettes. The reaction was monitored by recording the changes in the absorption spectrum of crocin at 440 nm.

Subsequently, the Fe‐catalyzed photo‐oxidation resistance test was conducted to assess the presence of active radicals. Crocin, crocin–AG, and crocin–Na_2_GA solutions with fixed crocin concentrations (0.1 and 0.01 mM) were prepared, and FeCl_3_ (0.033 mM) was added to each solution. The mixtures were irradiated under identical conditions, and the reaction was monitored by measuring the changes in the absorption spectrum of crocin at 440 nm.

### 2.5 Photoprotection of Carotenoids and Retinoids by Crocin–Na_2_GA

The photostability of the active compounds in aqueous solution was evaluated using a high‐light damage test in a drug high‐light stability test chamber (SHH‐GD‐2, YSEI, China). Water‐soluble retinol, astaxanthin, and β‐carotene samples were prepared at active ingredient concentrations of 0.5 mg/mL. Crocin–Na_2_GA was then added to each sample to obtain a crocin concentration of 0.3 mg/mL. All samples were sealed in transparent tubes (diameter = 2.5 cm) and exposed to LED light with an intensity of 4500 ± 500 lx at 25°C. The residual concentrations of retinol, astaxanthin, and β‐carotene (w/w %) were determined at 1, 2, 4, 8, 16, 24, 48, 72, and 120 h using HPLC analysis. Here, the maximum absorption wavelength of crocin (wavelength at 440 nm) was selected for this determination.

Further, a long‐term photoprotection experiment was conducted using retinol as the model compound. The retinol concentration in each sample was fixed at 0.5 mg/mL, while the ratio of crocin–Na_2_GA to retinol was varied by adjusting the amount of crocin–Na_2_GA added. The high‐light damage test was performed under identical experimental conditions, and the samples were analyzed by HPLC at 0.5, 1, 2, and 3 days. The retention of retinol in each sample was then calculated.

### 2.6 Electrochemical Characterization

All electrochemical measurements were conducted in a 100 mL three‐electrode single‐compartment cell containing a 100 mM Na_2_SO_4_ solution, controlled by an electrochemical workstation (CHI 650D, Chenhua Co. Ltd., China). A glassy carbon disk electrode (diameter: 3 mm) coated with the test materials, a platinum foil (20 × 30 mm), and an Ag/AgCl (3.0 M) electrode were used as the working, counter, and reference electrodes, respectively. The samples were dissolved in a 50 mM Na_2_SO_4_ solution, and the concentrations of crocin, retinol, astaxanthin, and β‐carotene were adjusted to 1 mg/L. Cyclic voltammetry (CV) curves were recorded within the potential range of 0.6 to −1.2 V vs. Ag/AgCl at a scan rate of 0.1 V/s. Electrochemical capacitance was determined by performing a series of CV scans from 0 to 0.1 V vs. Ag/AgCl at various scan rates (10, 20, 40, 60, 80, and 100 mV/s) (Figure S2).

### 2.7 Preparation of Cream‐Based Formulations

Astaxanthin or retinol (50 mg) was added to olive oil (8 mL) and stirred until completely dissolved. The resulting solution was stored away from light and used as the oil phase. Separately, 85 mL of purified water was measured to prepare the aqueous phase, followed by the addition of 5 mL of glycerin, 2 mL of the compound emulsifier SETHIGEL G57 (a commercially available cold‐process cosmetic emulsifier composed of sodium polyacrylate, 2‐ethylhexyl stearate, and trideceth‐6), and 25 mg of crocin–Na_2_GA (used as the photostabilizer). The control group was prepared in the same manner but without crocin–Na_2_GA. The oil phase was then added to the aqueous phase under light‐protected conditions and homogenized using a high‐speed shear homogenizer for 10 min until a uniform cream was formed. Based on these procedures, two types of formulations were prepared: creams containing astaxanthin or retinol with crocin–Na_2_GA, designated as ASX‐C‐OM and R‐C‐OM, respectively, and control creams without crocin–Na2GA, designated as ASX‐OM and R‐OM. These cream formulations were subsequently used for the photostability and skin model experiments.

### 2.8 Photostability of Astaxanthin and Retinol in Cream‐Based Formulations and Skin Models

Each cream sample was placed in a 10 × 10 cm transparent sealed bag, flattened, and sealed after air removal. The photostability test was performed according to the method described in **section 2.5**. On days 1, 3, 5, and 7, 1 g of each sample was collected and dissolved in 10 mL of anhydrous ethanol. After filtration, the concentrations of retinol, astaxanthin, and crocin on a particular storage day were compared with their initial concentrations to calculate their retention rates.

Artificial skin (VitroSkin, Florida Suncare Testing, USA) was used as a skin model. It is an advanced testing substrate that effectively mimics the surface properties of human skin. It contains both optimized protein and lipid components, which have been designed to have topography, pH, critical surface tension, and ionic strength similar to human skin. Prior to use, VitroSkin has to be placed for 24 h in a 14.85% w/w glycerin/water mixture for proper hydration. Then it was cut into square pieces with a side length of approximately 2.5 cm and divided into four groups. Each piece was treated with 1 mL of a cream sample and then placed in a light chamber with an intensity of 4500 ± 500 lx, a controlled temperature of 35°C± 0.5°C, and a relative humidity of 50% ± 10% to simulate skin conditions. After 24 h of exposure, the surface residual samples were collected using degreasing cotton swabs, and the surface layer of the VitroSkin was peeled off with adhesive tapes [29]. The tapes and cotton swabs used to collect the surface residual samples were immersed in 40 mL of methanol and treated in an ultrasonic water bath for 30 min. The extracts were filtered and analyzed by HPLC to quantify the surface contents (SCs) of retinol, astaxanthin, and crocin. The remaining VitroSkin samples were homogenized, and 40 mL of methanol was added to each homogenate to extract retinol and astaxanthin. Then, the extracts were analyzed by HPLC to quantify the compounds that had penetrated into the skin model, referred to as the penetration content (PC).

## 3 Results and Discussion

**FIGURE 1:**
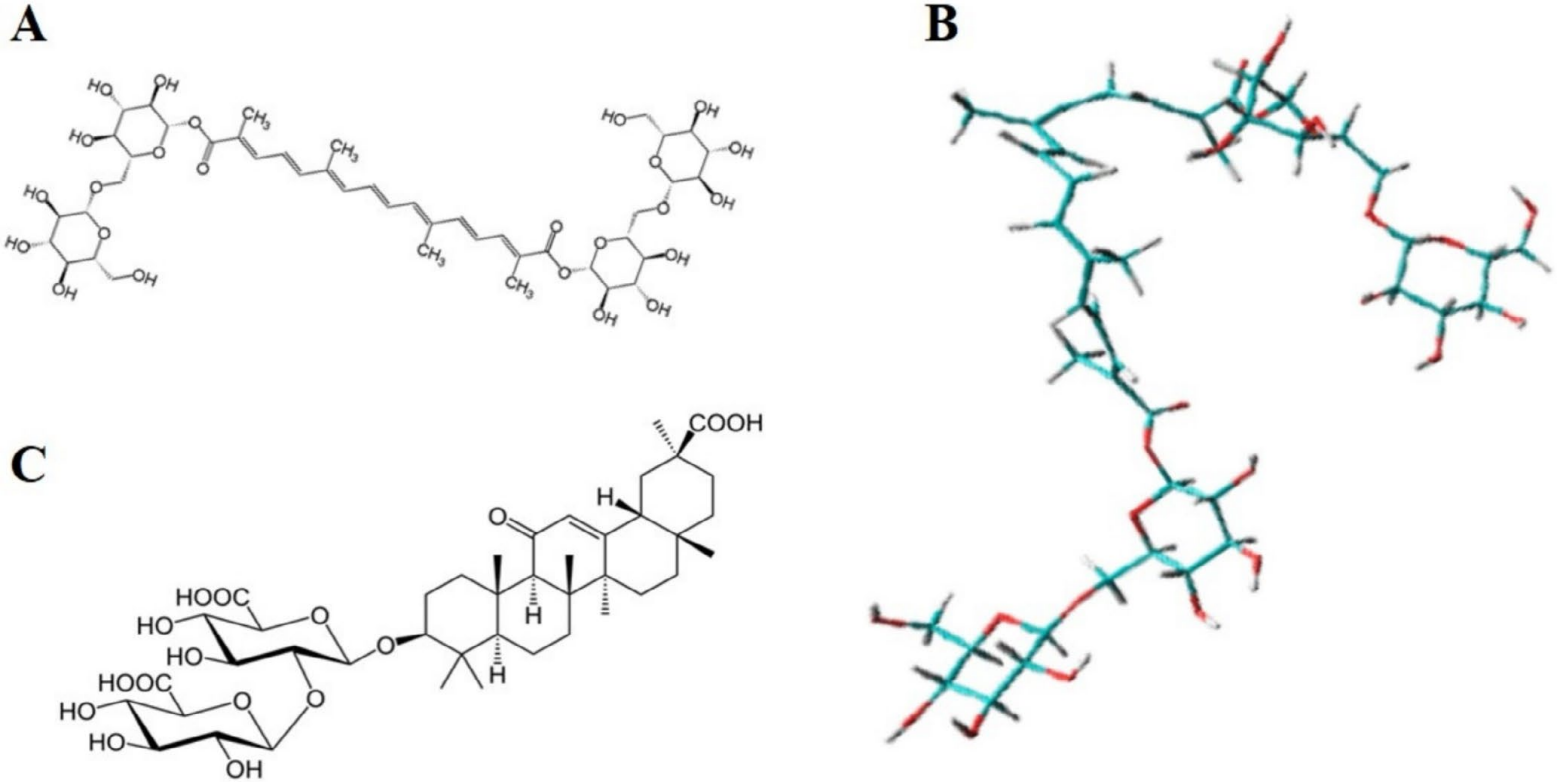
Molecular structure of Crocin (A), Crocin structure displayed in stick model after energy minimization in solution [31](B), and molecular structure of glycyrrhizic acid (C).

Crocin contains two sugar moieties (Figure 1A,B), and owing to its amphiphilic structure, it can form aggregates in aqueous solution [30, 31]. However, due to its molecular rigidity, these aggregates typically exist as parallel‐stacked oligomers, with the conjugated double‐bond regions remaining partially exposed. Based on previous findings, we hypothesized that mechanical treatment disrupts crocin aggregates, allowing crocin molecules to penetrate into the AG polymer matrix or form mixed nanoaggregates with Na_2_GA [10, 32, 33] (Figure 1C). Referring to these studies, we prepared crocin–Na_2_GA using a mechanochemical method [17]. For comparison, a crocin–AG complex was also prepared.

### 3.1 Stability of Crocin in Mixed Micellar Complexes

**FIGURE 2:**
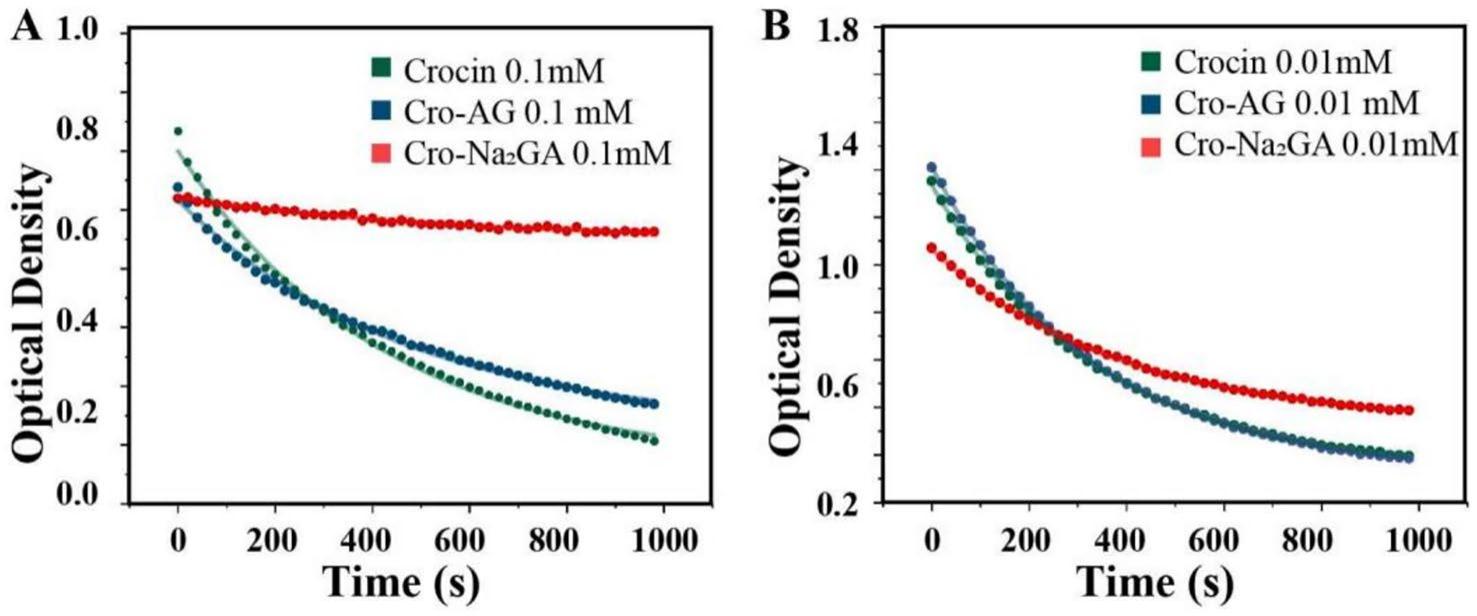
Fe‐induced oxidative degradation kinetics of Crocin in aqueous solution. (A) Degradation kinetics at 0.1 mM Crocin. (B) Degradation kinetics at 0.01 mM of Crocin.

To further examine the stability of the hybrid aggregates, the oxidative and photostability of the previously prepared crocin–Na_2_GA system were evaluated in aqueous solution. A comparable system, crocin–AG, prepared from crocin and the polysaccharide AG, was used as a control. The oxidative stability of crocin in both systems was first examined using Fe‐induced oxidation experiments conducted under oxygen‐rich conditions. The reaction kinetics were analyzed using an exponential decay model, and the first‐order reaction rate constants at 0.1 mM (Figure 2A) and 0.01 mM (Figure 2B) were obtained by fitting the data with the Leveneberg–Marquardt algorithm.

The results (Table 1) revealed a significant, concentration‐dependent decrease in the oxidation rate of crocin within the Na_2_GA‐containing mixed micelles, whereas only a slight effect was observed for the AG complex. We hypothesized that in polymer‐wrapped systems such as crocin–AG, the reduced crocin stability results from the rapid exchange between free crocin and the complex. Although this structure can provide partial protection against heat and oxygen, it cannot completely prevent proton interactions. In contrast, in the crocin–Na_2_GA mixed micellar system, the increased protective efficiency observed at higher crocin and Na_2_GA concentrations can be attributed to a shift in the equilibrium toward the micellar state. This phenomenon likely results from the low molecular weight of Na_2_GA, which enhances the encapsulation of crocin within the micelles. As a result, the exposure of crocin to the external environment is minimized, effectively preventing degradation induced by heat and oxygen.

**TABLE 1:** Rate constants of crocin oxidation induced by Fe(III) ions (1.0 mM FeCl_3_) in pure form and in mechanochemically prepared complexes.

System	Rate constant, in 10^−3^ s^−1^
0.01 mM Crocin	3.15 ± 0.02
0.01 mM Crocin–Na_2_GA (1:10)	2.57 ± 0.02
0.01 mM Crocin–AG (1:10)	3.04 ± 0.03
0.1 mM Crocin	2.37 ± 0.08
0.1 mM Crocin–Na_2_GA (1:10)	0.13 ± 0.01
0.1 mM Crocin–AG (1:10)	2.13 ± 0.08

### 3.2 Photosensitivity of Crocin in Mixed Micellar and Inclusion Complexes

Our previous studies have elucidated the mechanism of light‐catalyzed carotenoid oxidation [34, 35].

**FIGURE 3:**
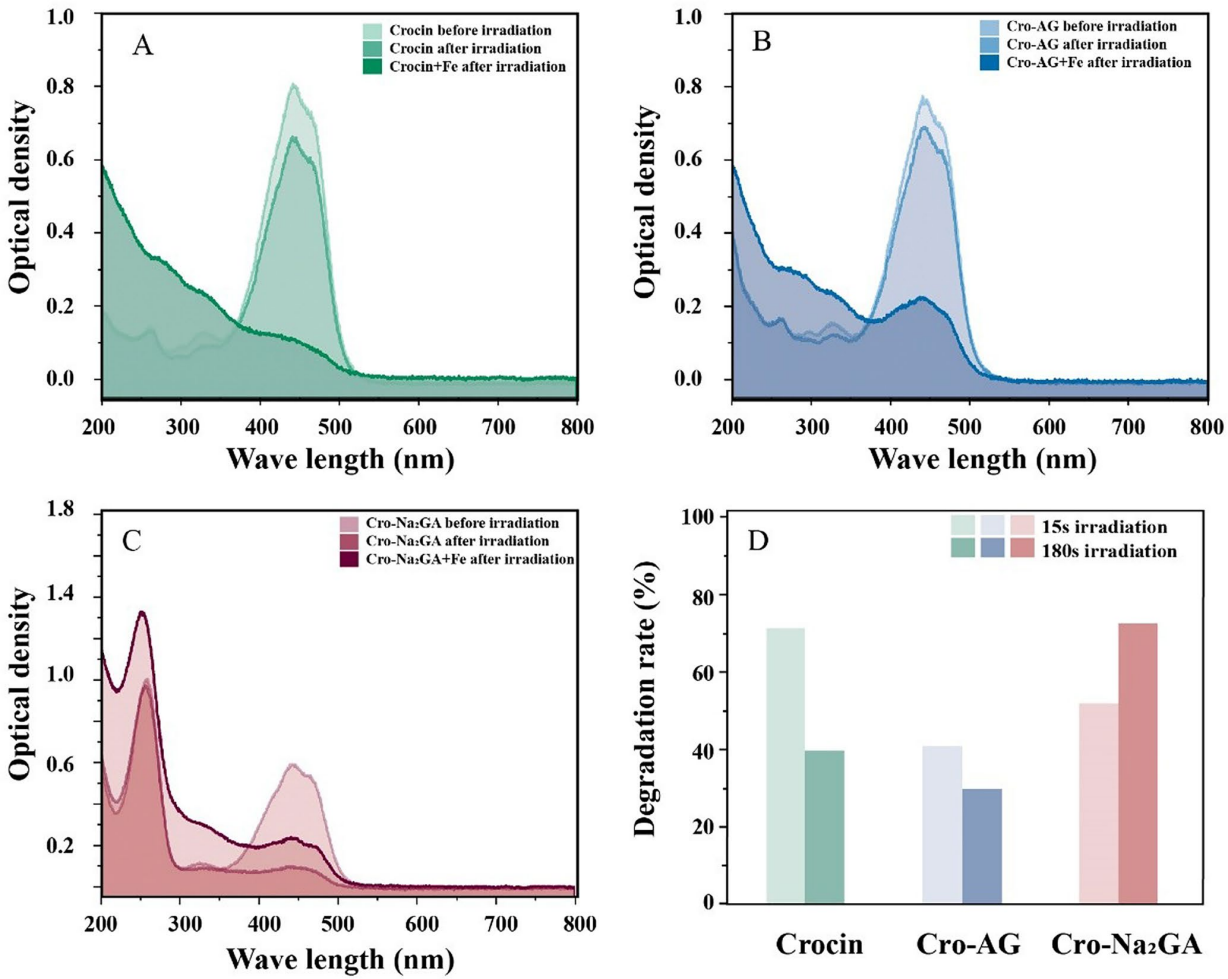
Photostability and Fe‐induced photodegradation of water‐solubilized Crocin in different forms. Optical density of Crocin (A), Crocin–AG (B), and Crocin–Na_2_GA (C) before and after laser irradiation, with or without the presence of 0.033 mM Fe^3+^. (D) Degradation degrees (%) of Crocin (0.01 mM) in different forms after irradiation: 15 s in the presence of Fe^3+^, and 180 s in the absence of Fe^3+^.

The process begins with electron transfer from a proton donor to the carotenoid, forming the corresponding radical cations. These cations are subsequently deprotonated into neutral carotenoid radicals, which are highly reductive and play an important role in photon absorption and photo‐radical quenching [36, 37]. This process is significantly accelerated in the presence of iron ions, as confirmed by ion‐catalyzed oxidation experiments. To elucidate the photostability of crocin–Na_2_GA, crocin–AG was used as a control representing a polymer‐wrapped crocin complex. Two light sources were employed: an excimer laser at 308 nm and a mercury lamp providing full‐spectrum light. Figure 3A shows the absorption spectra of a 0.01 mM aqueous crocin solution before and after 5 min of laser irradiation. When a catalytic amount of iron ions was added, visible discoloration occurred due to crocetin degradation, confirming the formation of photoinduced neutral carotenoid radicals.

Crocin in the crocin–AG complex exhibited only a 25% decrease in the photodegradation rate (Figure 3A,B), indicating that molecular‐level wrapping alone has a limited effect in improving crocin light stability. In contrast, crocin in the Na_2_GA‐containing mixed micellar system demonstrated a significant (> 90%) increase in the crocin photodegradation rate (Figure 3C,D). When Fe(III) was added to induce crocin photo‐oxidation, crocin–AG showed only a 2% decrease in crocin absorbance, whereas crocin–Na_2_GA exhibited a marked reduction of more than 44% after irradiation with full‐spectrum light from a mercury lamp. Similar trends were observed under 308 nm laser irradiation.

**SCHEME 1:**
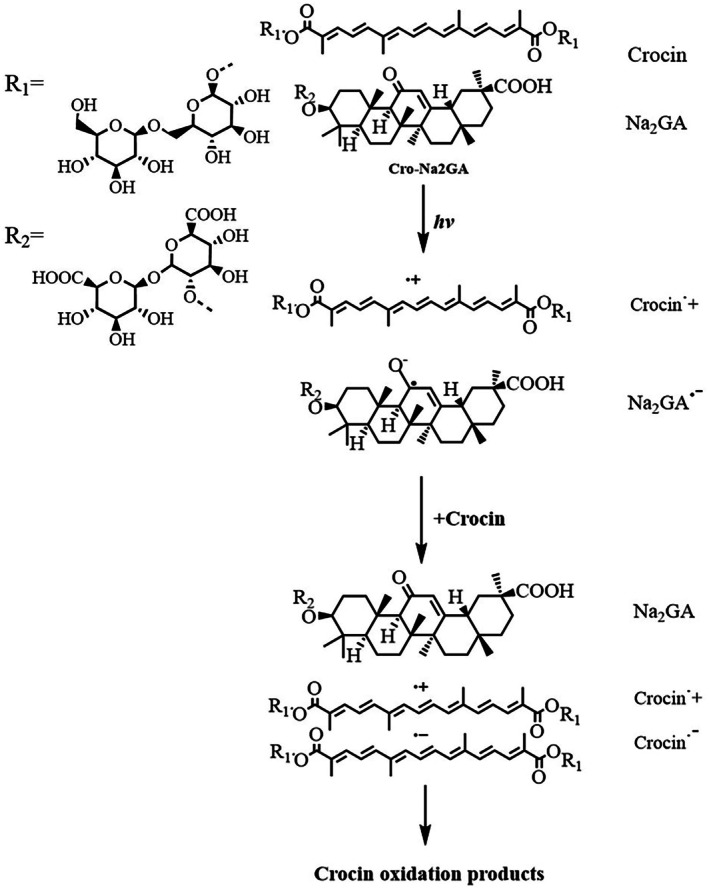
Electron transfer‐mediated photodegradation of crocin in the presence of Na_2_GA.

Given the recently reported ability of glycyrrhizinate (GA) to participate in proton‐coupled electron transfer (PCET) [38, 39], it is reasonable to infer that GA molecules promote the formation of neutral carotenoid radicals in aqueous systems. In addition, as reported by Edge et al. [40], carotenoid molecules can accept electrons from ketyl radical anions to form carotenoid radical anions [40], further contributing to the increased degradation rate of crocin in the crocin–Na_2_GA mixed micellar system (Scheme 1). Building on these findings, we investigated the potential of crocin–Na_2_GA as a photostabilizer for commercially available carotenoid‐ and retinoid‐based formulations.

### 3.3 Application of Crocin–Na_2_GA as a Photoprotective Agent

Crocin–Na_2_GA significantly enhanced the oxidative stability of crocin; however, it also increased the light sensitivity of the compound.

**FIGURE 4:**
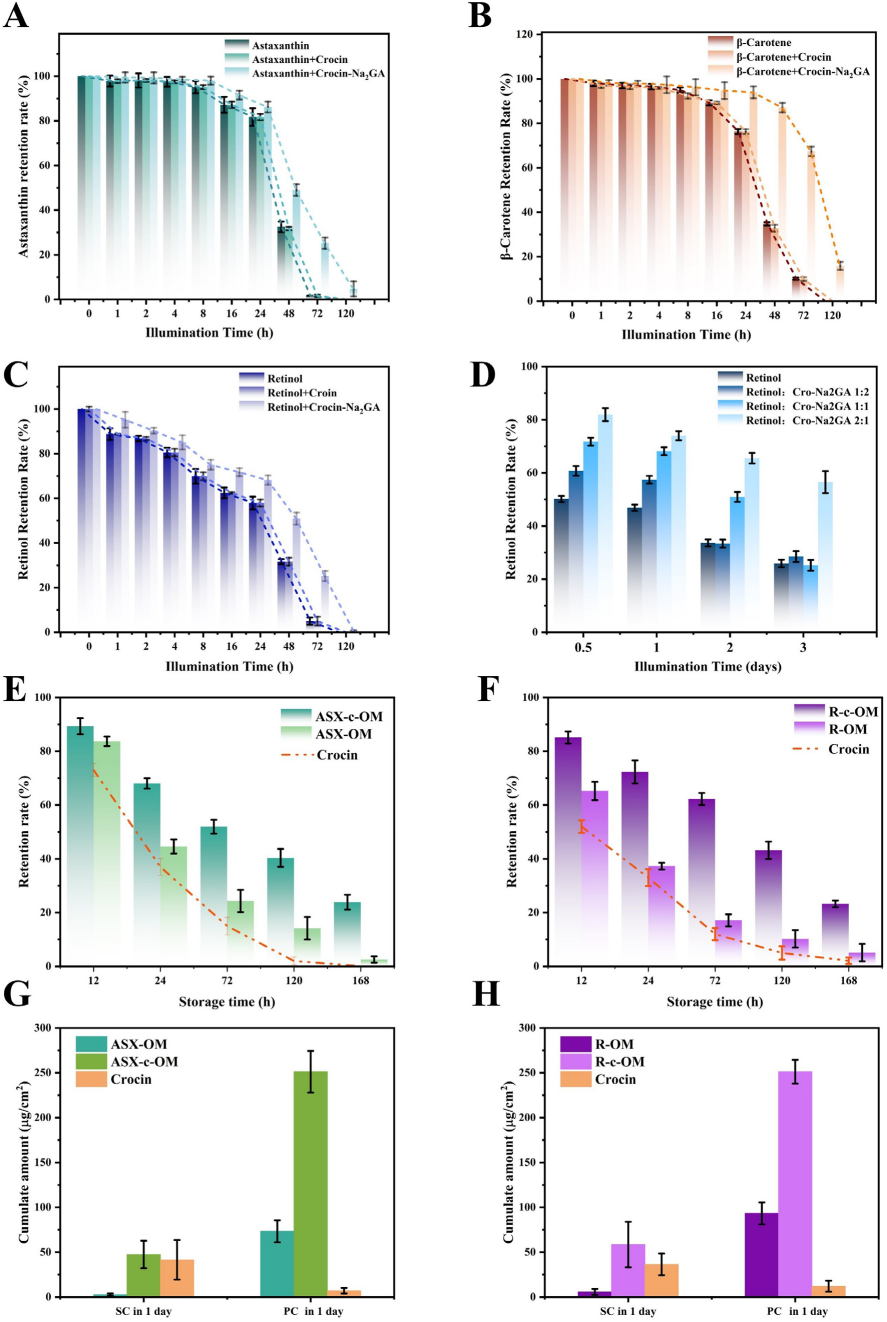
Color protection effect of Crocin–Na_2_GA on astaxanthin (A), β‐carotene (B), retinol (C), and retinol in different amounts of Crocin–Na_2_GA (D). Photoprotection effect of astaxanthin (E) and retinol (F) in the Cream product and on the skin model (G and H).

To evaluate the potential of crocin–Na_2_GA as a photoprotective agent, its effects on several naturally occurring photosensitive compounds, including retinol, β‐carotene, and astaxanthin, were examined in aqueous solution under strong light irradiation (Figure 4A–C). Compared to pure crocin, crocin–Na_2_GA showed markedly lower degradation rates of these active ingredients, improving their stability by 10%–50%, particularly under prolonged light exposure (> 8 h). For example, a comparable degradation level of β‐carotene (~10%) was observed after 16 h of irradiation in the absence of crocin–Na_2_GA and after 48 h in its presence. The long‐term (> 12 h) photoprotective effect at different ratios of crocin–Na_2_GA to retinol was further investigated (Figure 4D), revealing that a ratio of 1:2 at a fixed retinol concentration, especially after 72 h of exposure.

To verify the applicability of crocin–Na_2_GA in emulsion systems for pharmaceutical and cosmetic formulations containing carotenoids and retinol, creams incorporating retinol or astaxanthin were prepared with crocin–Na_2_GA added as a light stabilizer at the optimal concentration. The degradation rates of retinol and astaxanthin in these emulsions were then evaluated under simulated natural light conditions (Figure 4E,F). In the control creams, retinol and astaxanthin underwent rapid photodegradation, with their effective contents decreasing sharply to only 10% of their initial amounts after 24 h, consistent with their well‐known poor light stability. Upon the addition of crocin–Na_2_GA, the contents of retinol, astaxanthin, and crocetin (used as an indicator of crocin degradation) were measured. The crocetin content in the cream gradually decreased with extended irradiation, whereas the degradation of retinol and astaxanthin was significantly slowed. After 24 h, the retinol and astaxanthin contents remained at 72.5% and 63.2% of their initial values, respectively—approximately 6–7 times higher than those in the control group. These findings suggest that the crocin mixed micelles effectively scavenge photoinduced free radicals through a self‐sacrificial mechanism, thereby protecting the active ingredients.

The stabilizing effect of crocin mixed micelles on retinol and astaxanthin in cream‐based formulations was subsequently investigated under simulated daily application conditions. Creams containing retinol or astaxanthin were applied to the surface of pretreated artificial skin, and their stability was assessed. The SCs of retinol and astaxanthin increased significantly, by 10‐ to 17‐fold, after the addition of the light stabilizer (Figure 4G,H). Additionally, the PCs of these antioxidants increased by 2.5‐ to 3.5‐fold, indicating that crocin mixed micelles offer significant advantages as light stabilizers for antioxidant‐based pharmaceutical and cosmetic applications.

Overall, these results indicate that the antioxidant capacity of crocin mixed micelles is significantly enhanced compared to that of pure crocin. After illumination, crocin–Na_2_GA provides effective photodegradation protection for astaxanthin, β‐carotene, and retinol. This demonstrates that the system can be stably incorporated into aqueous formulations containing carotenoid‐type conjugated alkenes, which are prone to light‐induced degradation, thereby ensuring long‐term stability during storage and application.

### 3.4 Mechanistic Investigation Into the Role of Crocin–Na_2_GA as a Photoprotective Agent

Previous studies have reported that the dimerization of crocin in aqueous solution may lead to electron delocalization, weakening its free radical scavenging ability [41, 42]. For crocin to exhibit effective free radical scavenging activity, crocin–Na_2_GA should self‐assemble into a stable nanodispersion system in solution to prevent crocin self‐aggregation.

**FIGURE 5:**
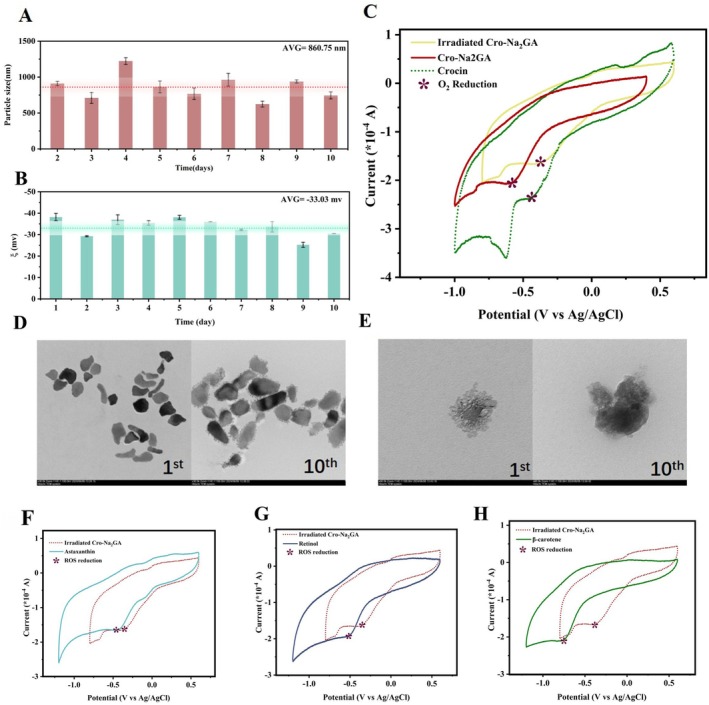
Particle size and zeta potential changes in the Crocin–Na_2_GA system over time. (A) Particle size distribution at 10 days. (B) Zeta potential exchanges at 10 days. (C) Redox potential measurements of Crocin, Crocin–Na_2_GA, and irradiated Crocin–Na_2_GA.(D) TEM graphic of Crocin–Na_2_GA at 15 K zoom magnification on day 1 and day 10. (E) TEM graphic of Crocin–Na_2_GA at 60 K zoom magnification on day 1 and day 10. Redox potential measurement of oxygen reduction in the presence of astaxanthin (F), retinol (G), and beta‐carotene (H).

To verify the effect of crocin–Na_2_GA on crocin self‐polymerization, the molecular weight distribution of crocin aggregates was investigated. Pure crocin solution displayed peaks around 141, 82, 36, and 5 kDa (Figure S1B). In contrast, when a 0.5 M LiNO_3_ solution was used to suppress self‐association [43], only a single monomeric peak was observed at < 1 Da (Figure S1A), indicating that crocin exists as a multimer in aqueous media. However, when crocin was mixed with Na_2_Ga in solution, the peak shifted to 171 kDa, differing from those observed for pure Na_2_GA (100 kDa) or pure crocin (Figure S1C,D). This finding suggests the formation of complexes between Na_2_GA and crocin, with crocin self‐polymerization being completely inhibited. The particle stability of the crocin–Na_2_GA system was subsequently investigated. During long‐term storage, crocin–Na_2_GA particles remained highly stable in solution, with an average particle size of 860.75 nm (Figure 5A) and a zeta potential of −33.03 mV (Figure 5B) over 10 days, demonstrating that the system exists on the nanoscale and maintains long‐term stability in solution. TEM analysis (Figure 5D,E) revealed that the nanoparticles exhibited a clustered bundle‐like morphology that showed no obvious changes after 10 days. These results confirm that the crocin–Na_2_GA system maintains good stability in solution without further crocin self‐aggregation during prolonged storage.

Decomposition of carotenoids and retinol in aqueous solution under light exposure mainly results from light‐induced redox reactions. Therefore, the effect of crocin–Na_2_GA on the photocatalytic redox behavior of crocin was investigated. Electrochemical techniques have recently become reliable tools for qualitative and quantitative studies of redox processes, as redox potential measurements can help elucidate reaction pathways and transition states [44, 45]. According to general redox principles, in a complex system containing multiple reducing agents, those with lower reduction potentials (in absolute value) preferentially undergo oxidation. Only after their depletion do substances with higher potentials react [46]. Thus, the oxidation sequence depends on the composition of the system. In this study, potential measurements were used to explain the superior photoprotective performance of crocin–Na_2_GA.

CV measurements for oxygen reduction of crocin and crocin–Na_2_GA showed that the reduction potential of crocin (marked as *, absolute value) increased from 0.42 to 0.56 V in crocin–Na_2_GA under dark conditions but decreased to 0.37 V after light irradiation (Figure 5C). These results indicate that in the absence of light, Na_2_GA enhances crocin stability by forming a supramolecular system more stable than pure crocin, consistent with the results of photostability experiments. However, upon light exposure, the micellar supramolecular complex induces crocin to form more reactive intermediates with stronger reducing properties.

The redox potentials of astaxanthin (Figure 5F), retinol (Figure 5G), and β‐carotene (Figure 5H) in the presence of crocin–Na_2_GA were then investigated, showing values of 0.46, 0.47, and 0.75 V, respectively. These potentials were lower than that of crocin–Na2GA in the dark (0.56 V) but higher than that of crocin–Na_2_GA under illumination (0.37 V). This finding suggests that the photoinduced oxidative degradation of crocin within crocin–Na_2_GA occurs before the photodegradation of astaxanthin, β‐carotene, and retinol, allowing these active compounds to be effectively protected in the presence of crocin–Na_2_GA. These findings are consistent with the previously observed rapid degradation of astaxanthin and retinol after 24 h in photostability experiments.

Overall, the experimental results demonstrate that crocin–Na_2_GA first forms a stable nanomicellar supramolecular system through self‐assembly in solution. By interacting with crocin via van der Waals forces, Na_2_GA inhibits crocin self‐polymerization, stabilizing the molecule and maintaining the exposure of its conjugated double bonds under dark conditions. Electrochemical results further suggest that upon light exposure, Na_2_GA acts as a radical transporter within the crocin–Na_2_GA system (Scheme 1), significantly lowering the redox potential of crocin, lower than that of astaxanthin, one of the strongest natural reducing agents. This enables crocin to preferentially quench photoinduced radicals, thereby effectively protecting the active components from photodegradation.

## 4 Conclusion

Carotenoids and retinol are essential active ingredients in pharmaceuticals and cosmetics. However, their applications have been limited by poor photostability. Although their light resistance can be improved to some extent by constructing supramolecular complexes or adding light stabilizers, the safety and regulatory approval of these materials for use in drugs, cosmetics, and food products remain insufficient. In this study, the innovative application of crocin–Na_2_GA nanocomposite micelles as self‐sacrificial photostabilizers for carotenoid‐ and retinol‐containing formulations was demonstrated, building upon previous serendipitous findings regarding the construction of crocin supramolecular systems. Crocin–Na_2_GA mixed micelles were more stable than pure crocin in the absence of light but became highly active when exposed to light, effectively protecting various carotenoids and retinol from photodegradation and thereby preserving their biological activity. Crocin–Na_2_GA could be incorporated into aqueous or emulsion systems at an additive concentration of 2.5%, achieving up to a 50% reduction in the photodegradation of carotenoids and retinol during storage and at least a 10‐fold improvement during skin application. Mechanistic studies revealed that crocin–Na_2_GA formed a stable nanomicellar system. In this system, Na_2_GA inhibited crocin self‐polymerization, while light exposure and possible radical transformation of Na_2_GA induced crocin to generate radicals with higher reduction potentials compared to astaxanthin, β‐carotene, and retinol. Consequently, crocin was preferentially consumed instead of these active molecules. In conclusion, this study demonstrates that crocin–Na_2_GA is a safe and effective photostabilizer for carotenoids and retinol, meeting the requirements for applications in pharmaceuticals, cosmetics, and foods.

## Author Contributions

W. Xu, N.E. Polyakov, and A.V. Dushkin: writing – review and editing, supervision, resources, methodology, conceptualization, funding resources. M. Zhao, S. Chen, and K. Hu: writing – original draft, formal analysis, visualization, investigation. V.I. Evseenko, E.S. Meteleva, M.V. Zelikman, A.V. Mastova, M.A. Ulyanova, P.A. Kononova, O.Yu. Selyutina: writing – original draft, visualization, investigation, formal analysis, data curation.

## Funding

This work was supported by the National Key Research and Development Program of China 2021YFC2101005 and the Russian Federal Ministry of Science and Higher Education FWGF‐2021‐0003, 121032500061‐7.

## Ethics Statement

The authors have nothing to report.

## Consent

The authors have nothing to report.

## Conflicts of Interest

The authors declare no conflicts of interest.

## Supporting information


**Table S1:** Optimal Milling time of crocin system made by mechanochemistry Fomulation Milling time Retention rate of crocin (w/w %)
**Figure S1:** The SEC chromatography spectra of 0.02% wt% crocin water, in 0.2% LiNO3 (A), in 0.2% NaN3 water solutions (B), Na2GA in 0.2% LiNO3 (C), and a mixture Na2GA/crocin in a mass relation = 1/3 in 0.2% LiNO3 (D) solution.
**Figure S2:** CVs of astaxanthin (A), retinol (B), and beta‐carotene (C), crocin (D), crocin–Na2GA (E), and irradiated crocin–Na2GA (F), in water solution.

## Data Availability

The data that support the findings of this study are available from the corresponding author upon reasonable request.
