# Supplementary material for: Development of Efficient Supramolecular Photostabilizer for Carotenoids and Retinoids: Analyses and Application Research
Source: J Cosmet Dermatol. 2026 Jan 2;25(1):e70650. doi: 10.1111/jocd.70650 (PMC12759200; doi:10.1111/jocd.70650)
Supplement: Supplementary file 1 — Table S1: Optimal Milling time of crocin system made by mechanochemistry Fomulation Milling time Retention rate of crocin (w/w %) Figure S1: The SEC chromatography spectra of 0.02% wt% crocin water, in 0.2% LiNO3 (A), in 0.2% NaN3 water solutions (B), Na2GA in 0.2% LiNO3 (C), and a mixture Na2GA/crocin in a mass relation = 1/3 in 0.2% LiNO3 (D) solution. Figure S2: CVs of astaxanthin (A), retinol (B), and beta‐carotene (C), crocin (D), crocin–Na2GA (E), and irradiated crocin–Na2GA (F), in water solution. [file JOCD-25-e70650-s001.docx]

Support Information

**Development of efficient supramolecular photo-stabilizer for carotenoids and retinoids: analyses and application research**

**1. Preparation of crocin complex**

The crocin and the auxiliary material Na_2_GA are placed in a 300 mL cylindrical Teflon ball mill tank at a mass ratio of crocin/auxiliary equal 1/10. After adding steel balls (diameter 22 mm, load 675 g), use a roller ball mill VM-1 prepares crocin complex at an acceleration of 1 g and a rotation speed of 156 rpm. The ball milling duration was 8 hours, and samples were taken at 2, 4, and 8 hours for optimal milling time. The optimal milling time was fixed as 2 h with the best retention rate of crocin. Samples made on this condition was named as Crocin-Na_2_GA.

**Table S1：Optimal Milling time of crocin system made by mechanochemistry**

| Formulation | Milling time | Retention rate of crocin (w/w %) |
| --- | --- | --- |
| Crocin-Na_2_GA-2 | 2 | 68.9 |
| Crocin-Na_2_GA-4 | 4 | 64.5 |
| Crocin-Na_2_GA-8 | 8 | 65.7 |
| Crocin-AG-2 | 2 | 61.9 |
| Crocin-AG-4 | 4 | 65.7 |
| Crocin-AG-8 | 8 | 62.6 |

Similar ratio and mill condition was carried out by using crocin and AG (Arabinogalactan) to prepare warping super molecular system as control. The ball milling duration was 8 hours, and samples were taken at 2, 4, and 8 hours for optimal milling time. The result of retention rate of crocin showed that optimal milling time was 4 h. Samples made on this condition was named as Crocin-AG.

**2. Molecular weight distribution**

Samples were dissolved in an aqueous solution of 0.2% NaN_3_ or LiNO_3_, Measurement of molecular weight distribution through size exclusion HPLC at a flow rate of 1mL/min and a temperature of 30°C. Results were calibrated using standard dextran with molecular weights of 1, 5, 12, 25, 80, 150, 270, and 410 kDa and processed using the Agilent GPC data analysis program (Santa Clara, CA).


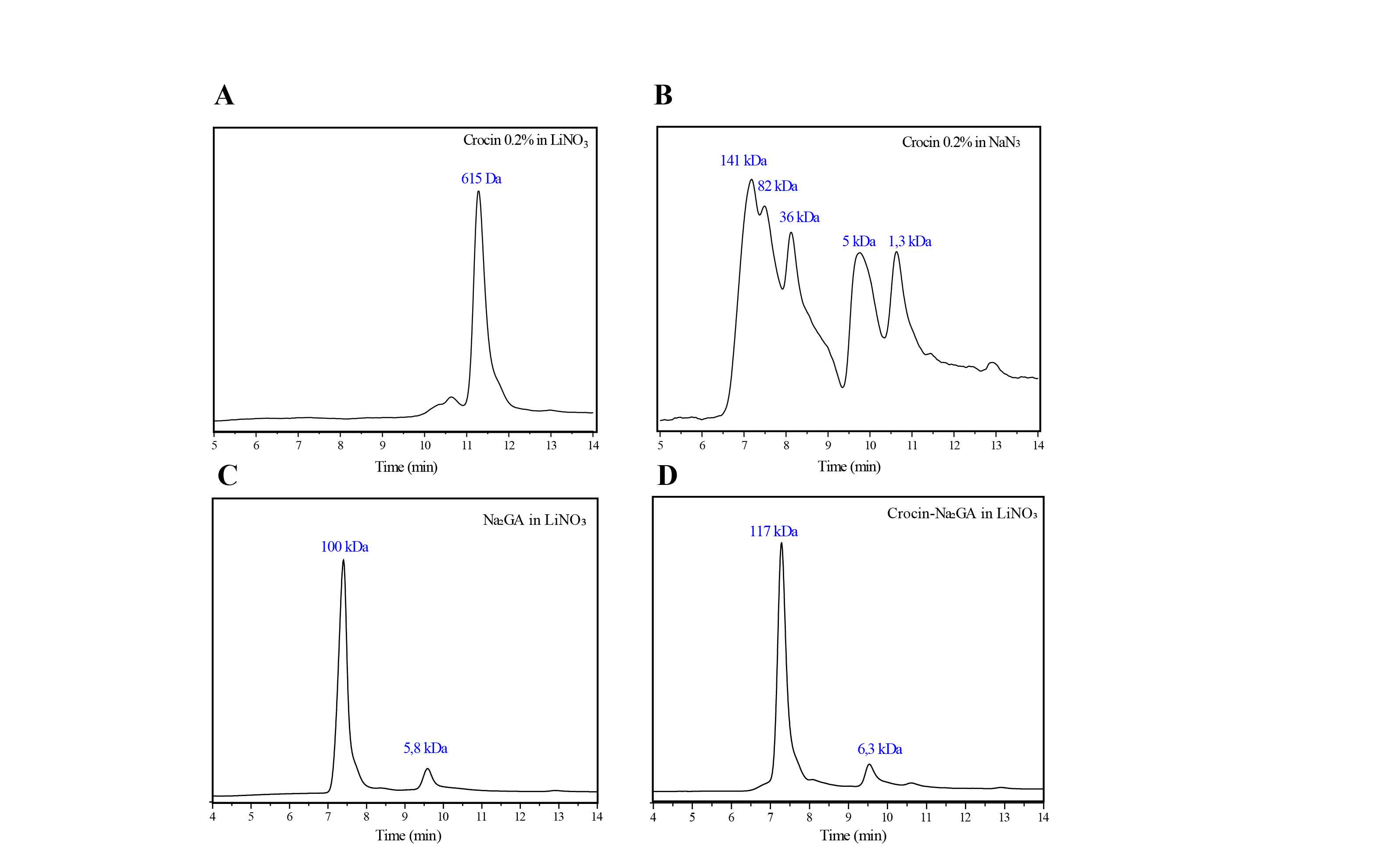


**Figure S1:** The SEC chromatography spectra of 0.02% wt% crocin water, in 0.2% LiNO_3_ (A), in 0.2% NaN_3_ water solutions (B), Na_2_GA in 0.2% LiNO_3_ (C) and mixture Na2GA/crocin in mass relation = 1/3 in 0.2% LiNO_3_ (D) solution.

1. **Cyclic voltammetry studies of samples**

All the electrochemical characterization was performed inside a 100 mL three-electrode single-compartment cell filled with 100 mM Na_2_SO_4_ solution, which was controlled by an electrochemical workstation (CHI 650D, Chenhua Co., China). A glassy carbon disk (diameter: 3 mm) coated with electrode materials, a platinum foil (20, 30 mm), and an Ag/AgCl (3.0 M) were used as the working electrode, the counter electrode, and the reference electrode, respectively. Samples were soluted into 50mM Na_2_SO_4_ solution and set the concentration of Crocin, retinol, astaxanthin, and β-carotenoids to 1ml/L. Cyclic voltammetry (CV) curves were recorded within the range from 0.6 to −1.2 V vs Ag/AgCl at a scan rate of 0.1 V s^−1^. Electrochemical capacitance was performed by running a series of CVs from 0 to 0.1 V vs Ag/AgCl, with different scan rates (10, 20, 40, 60, 80, 100 mV s^−1^). Cyclic voltammetry (CV) curves were shown below in Figure. S2.


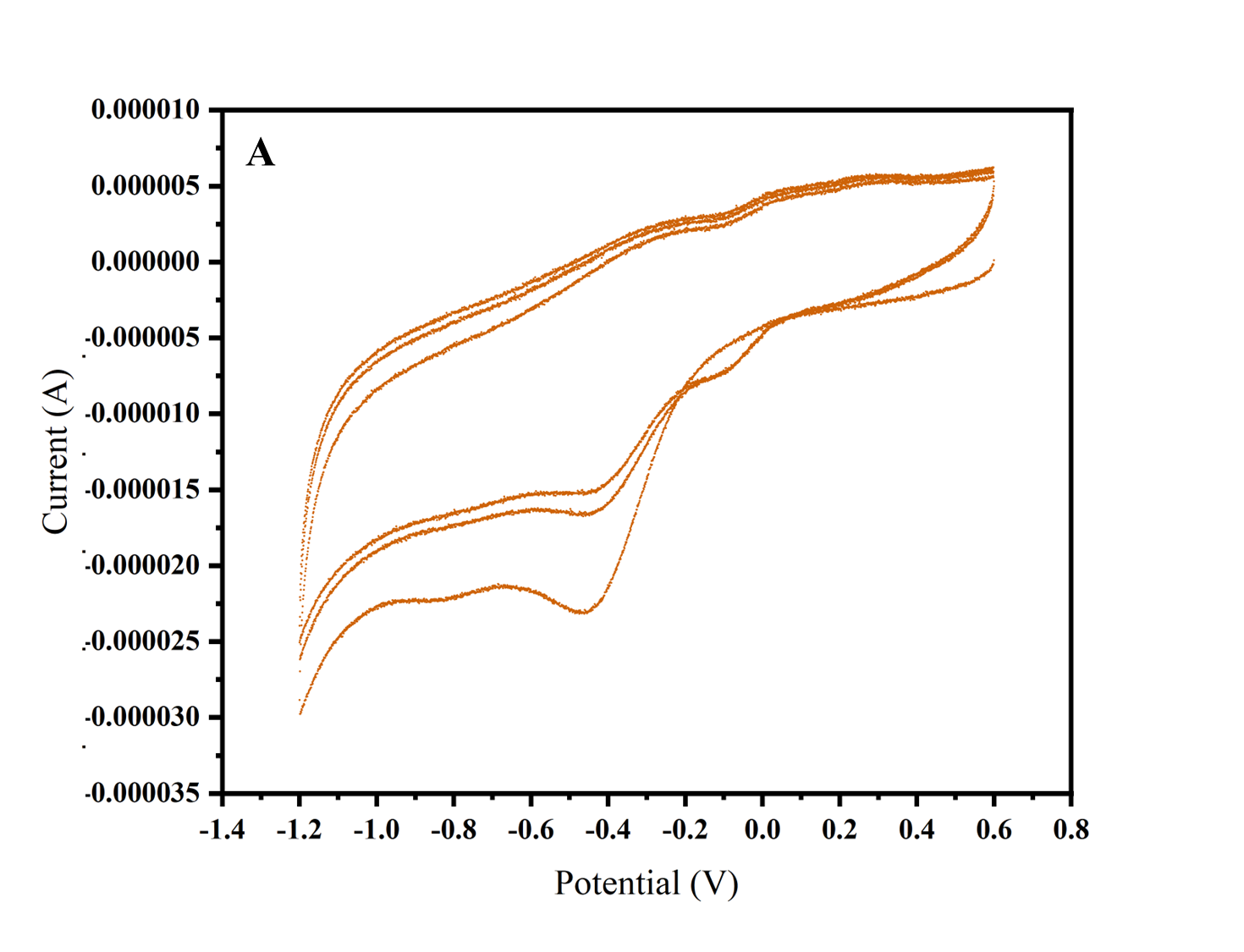

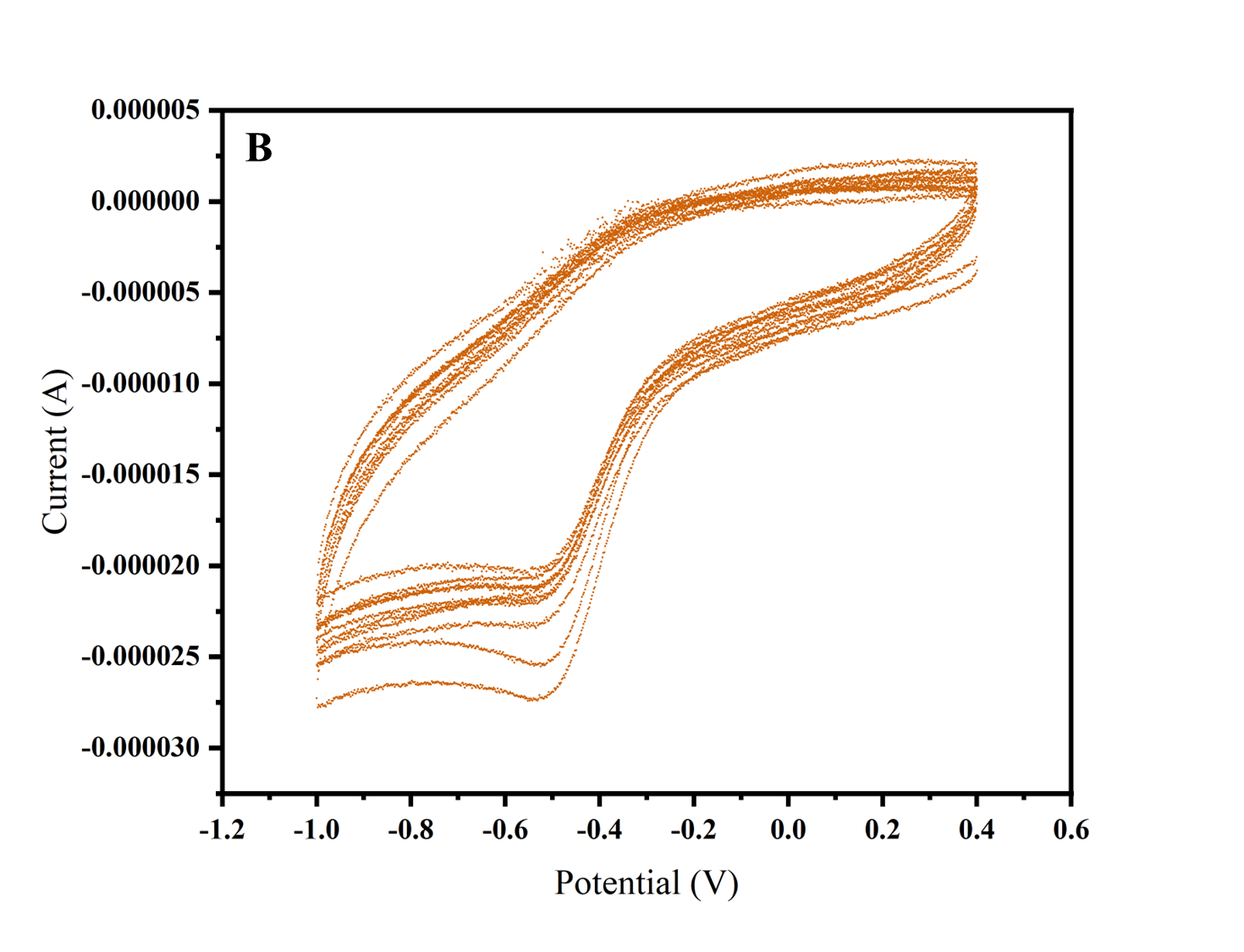


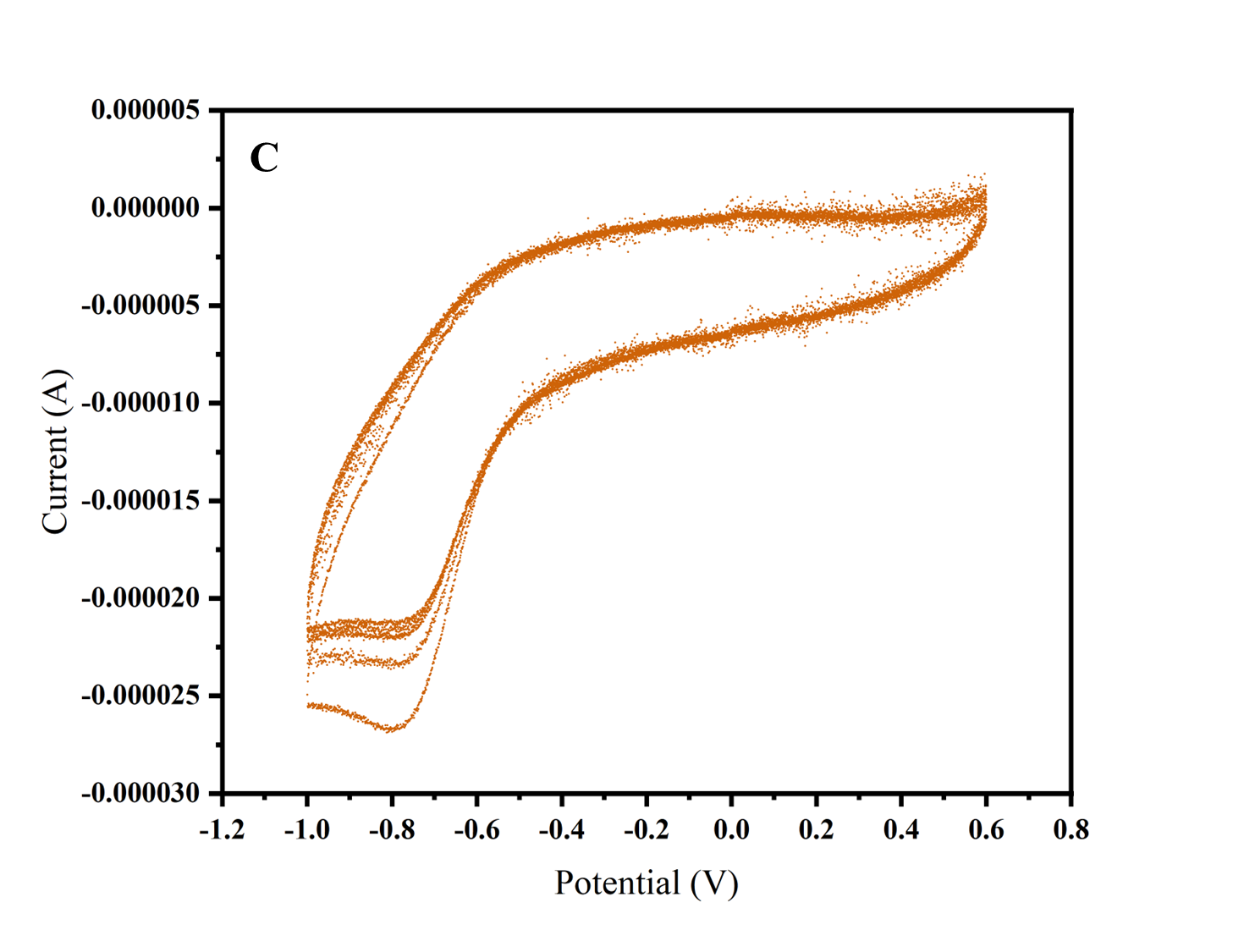

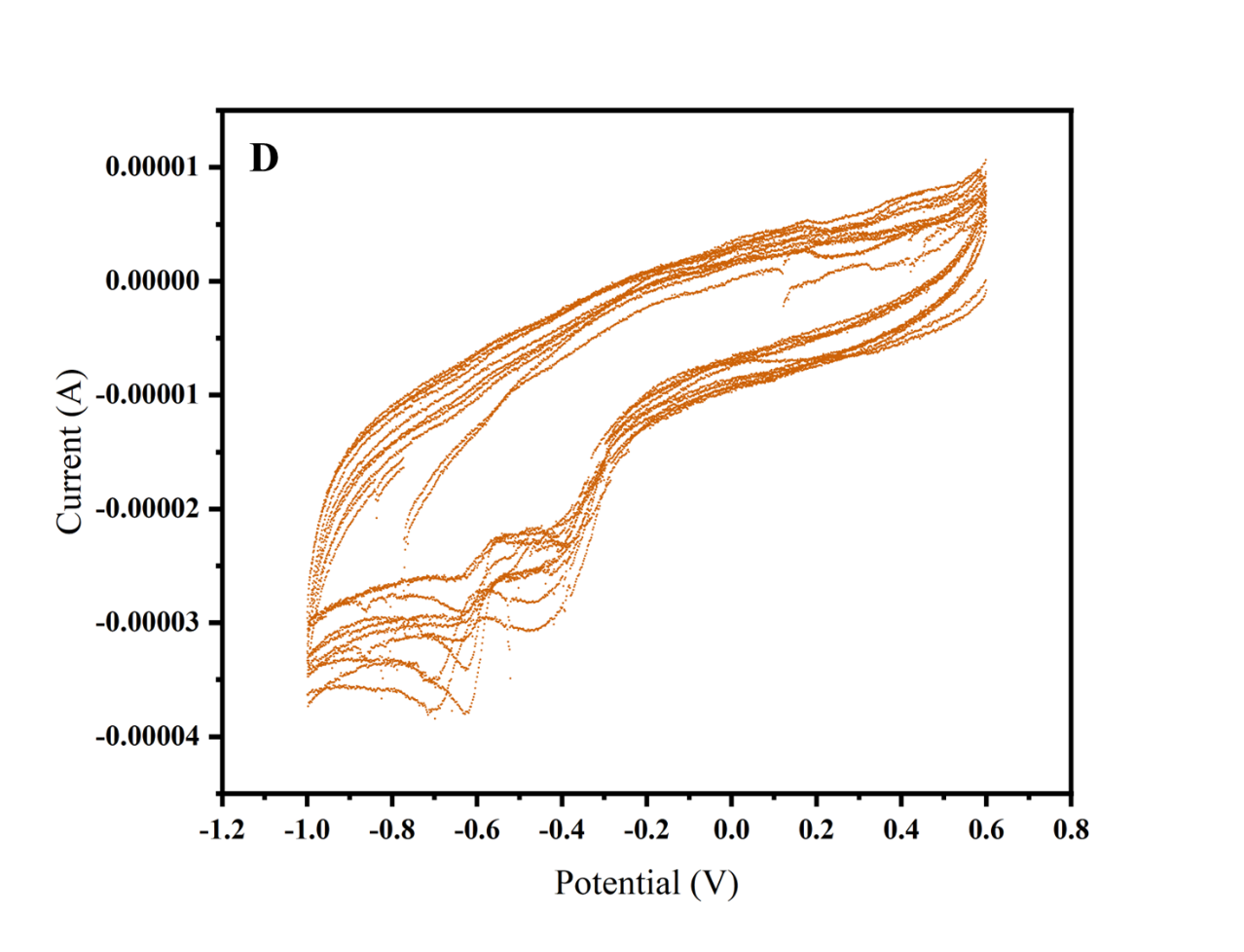


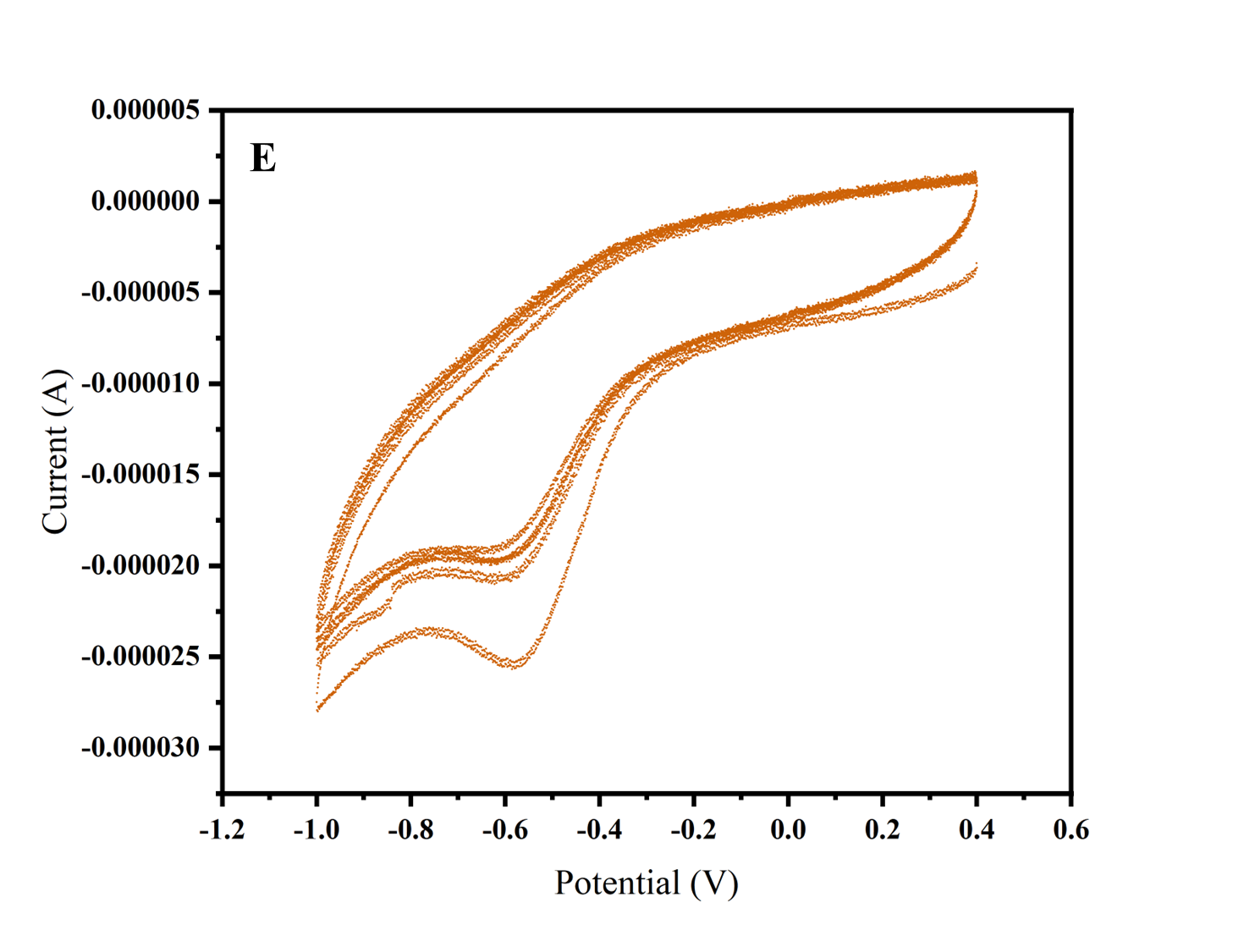

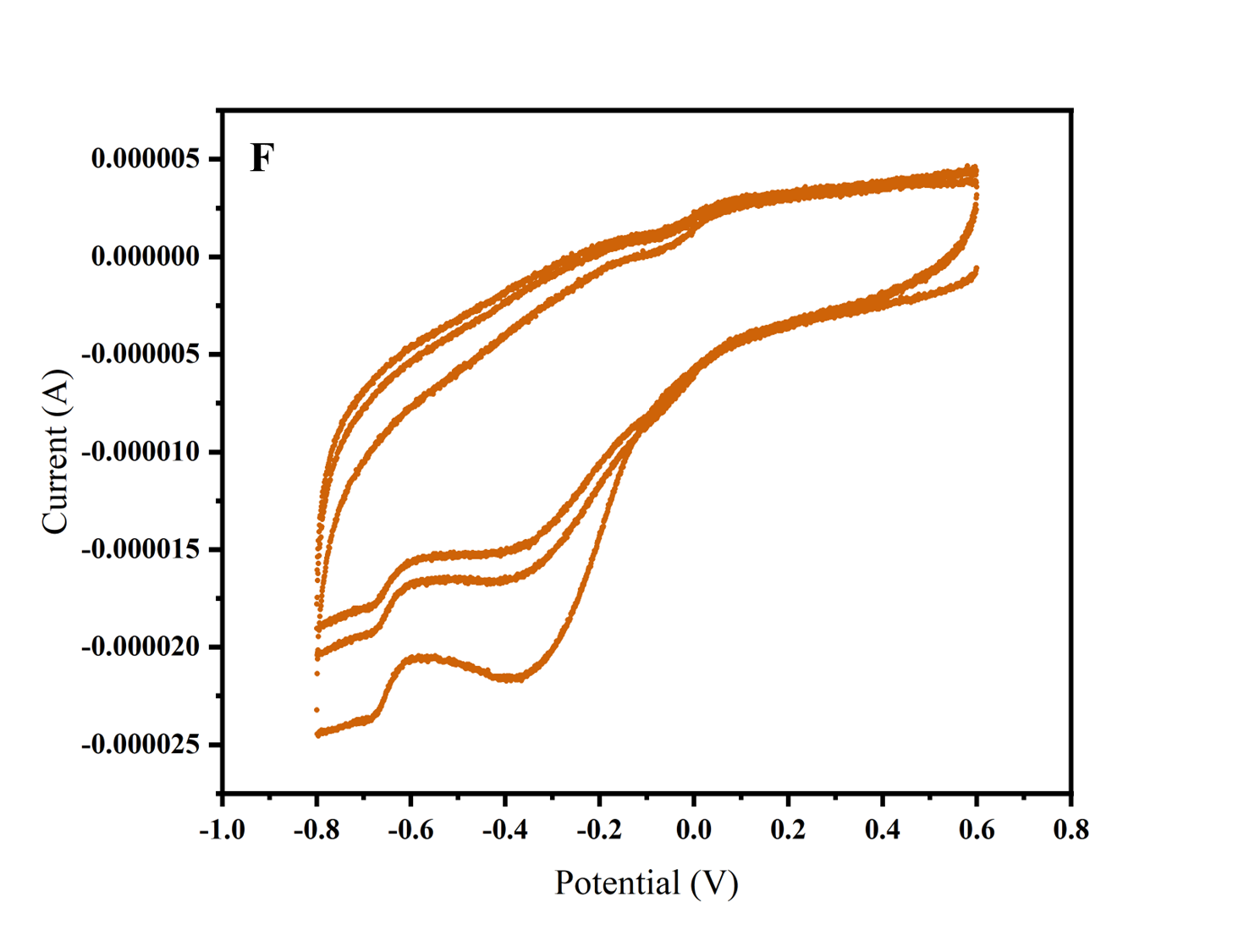


**Figure S2**: CVs of astaxanthin (A), retinol (B), and beta-carotene (C), Crocin (D), Crocin-Na_2_GA (E) , and irradiated Crocin-Na_2_GA(F) in water solution.
